# Supplementary material for: Targeted immunotherapy against distinct cancer-associated fibroblasts overcomes treatment resistance in refractory HER2+ breast tumors
Source: Nat Commun. 2022 Sep 9;13:5310. doi: 10.1038/s41467-022-32782-3 (PMC9463158; doi:10.1038/s41467-022-32782-3)
Supplement: Supplementary file 1 — Supplementary Information [file 41467_2022_32782_MOESM1_ESM.pdf]

Supplementary information for:

Targeted immunotherapy against distinct cancer-associated fibroblasts overcomes treatment resistance in refractory HER2+ breast tumors

Elisa I. Rivas[1#], Jenniffer Linares[1#], Melissa Zwick[1], Andrea Gomez-Llonin[1], Marc Guiu[2], Anna Labernadie[3], Jordi Badia-Ramentol[1], Ana Llado[2], Lidia Bardia[2], Ivan Perez-Nuñez[1], Carolina Martinez-Ciarpaglini[4], Noelia Tarazona[4], Anna Sallent-Aragay[1], Marta Garrido[1], Antoni Celia-Terrassa[1], Octavio Burgues[4], Roger R. Gomis[2], Joan Albanell[1,5,6,7†], Alexandre Calon[1†]*.

[1] Hospital del Mar Medical Research Institute (IMIM), Barcelona, Spain

[2] Institute for Research in Biomedicine (IRB), Barcelona, Spain

[3] Institute for Bioengineering of Catalonia (IBEC), Barcelona, Spain

[4] Instituto de Investigación Sanitaria (INCLIVA), Valencia, Spain

[5] Medical Oncology Department, Hospital del Mar, Barcelona, Spain.

[6] Universitat Pompeu Fabra, Barcelona, Spain.

[7] Centro de Investigación Biomédica en Red de Oncología (CIBERONC-ISCIII), Madrid, Spain.

Footnotes

[#] These authors contributed equally

[†] These authors jointly supervised this work

*Correspondence: acalon@imim.es

Supplementary Figure 1

Patient-derived breast fibroblasts and naïve ICs support BCCs expansion

Supplementary Figure 2

Response to IL2 anti-cancer immunity

Supplementary Figure 3

FAP-IL2v restores anti-cancer immunity

Supplementary Figure 4

FAP-IL2v enhances ADCC through NK activation

Supplementary Figure 5

FAP-IL2v enhances ADCC through NK activation

Supplementary Figure 6

FAP is a biomarker of TGF-beta activity in BC microenvironment

Supplementary Table 1

Clinical data from patients treated with anti-HER2-based therapy

Supplementary Table 2

Antibodies

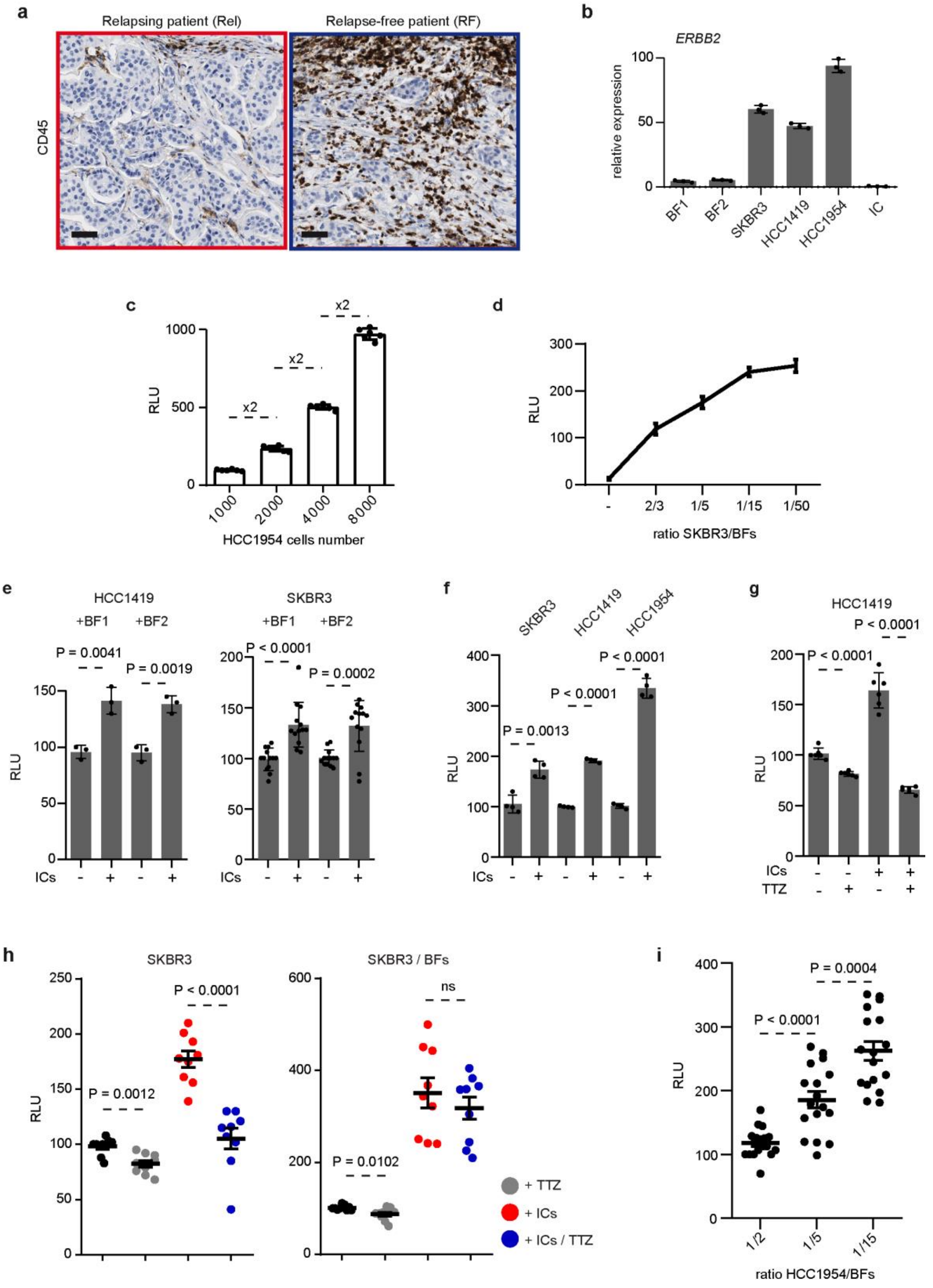
a
Relapsing patient (Rel)
Relapse-free patient (RF)
CD45
b
ERBB2
relative expression
BF1
BF2
SKBR3
HCC1419
HCC1954
IC
c
x2
RLU
HCC1954 cells number
1000
2000
4000
8000
d
RLU
ratio SKBR3/BFs
2/3
1/5
1/15
1/50
e
HCC1419
+BF1 +BF2
P = 0.0041
P = 0.0019
SKBR3
P < 0.0001
P = 0.0002
RLU
ICs
f
SKBR3
HCC1419
HCC1954
P = 0.0013
P < 0.0001
P < 0.0001
g
HCC1419
P < 0.0001
P < 0.0001
TTZ
h
SKBR3
P = 0.0012
P < 0.0001
SKBR3 / BFs
ns
P = 0.0102
+ TTZ
+ ICs
+ ICs / TTZ
i
P < 0.0001
P = 0.0004
ratio HCC1954/BFs
1/2
1/5
1/15

Supplementary figure 1. Patient-derived breast fibroblasts and naïve ICs support BCCs expansion. (a) Immunostaining of CD45+ cells in HER2+ BC human tumor samples from figure 1a. Representative of n=22 tumors. Scale bars: 50 µm. **(b)** qRT-PCR measuring relative expression levels of HER2 gene (*ERBB2*) in the indicated cells cultured *in vitro*. n=3 replicates from 1 out of 2 biologically independent experiments. Values are mean ± s.d. **(c)** Bioluminescence quantification is proportional to the amount of seeded HCC1954. Fold changes between luminescence measurements are indicated. n=6 biologically independent experiments. Values are mean ± s.d. **(d)** Bioluminescent tracking of SKBR3 co-cultured with increasing amount of BFs. n=3 biologically independent experiments. Values are mean ± s.d. **(e)** Bioluminescent tracking of HCC1419 (n=3) and SKBR3 (n=13) in HER2+3DiBCs from 5 biologically independent experiments. Values are mean ± s.d. **(f)** Bioluminescent tracking of indicated BCCs in HER2+3DiBCs cultured w/o ICs. n=4 HER2+3DiBCs examined per condition, from 2 biologically independent experiments. Values are mean ± s.d. **(g)** Bioluminescent tracking of HCC1419 in HER2+3DiBCs with ICs, TTZ or ICs+TTZ compared to control (IgG1). n=6 HER2+3DiBCs examined per condition, from 2 biologically independent experiments. Values are mean ± s.d. **(h)** Bioluminescent tracking of SKBR3 in HER2+3DiBCs with ICs (red), TTZ (gray) or ICs+TTZ (blue) in absence (left panel) or in presence of BFs (right panel). IgG1 was used as control for TTZ. n=9 HER2+3DiBCs examined per condition, from 3 biologically independent experiments. Values are mean ± s.e.m. **(i)** Bioluminescent tracking of HCC1954 in HER2+3DiBCs with increasing amount of BFs and treated with TTZ in presence of ICs. n=17 HER2+3DiBCs examined per condition, from 4 biologically independent experiments. Values are mean ± s.e.m. BF: breast fibroblasts; IC: immune cells; TTZ: trastuzumab; RLU: relative luminescence units. Two-sided, unpaired t-test *p*-values (P) are indicated for (e-i); ns indicates non-significant. Source data are provided as a Source Data file.

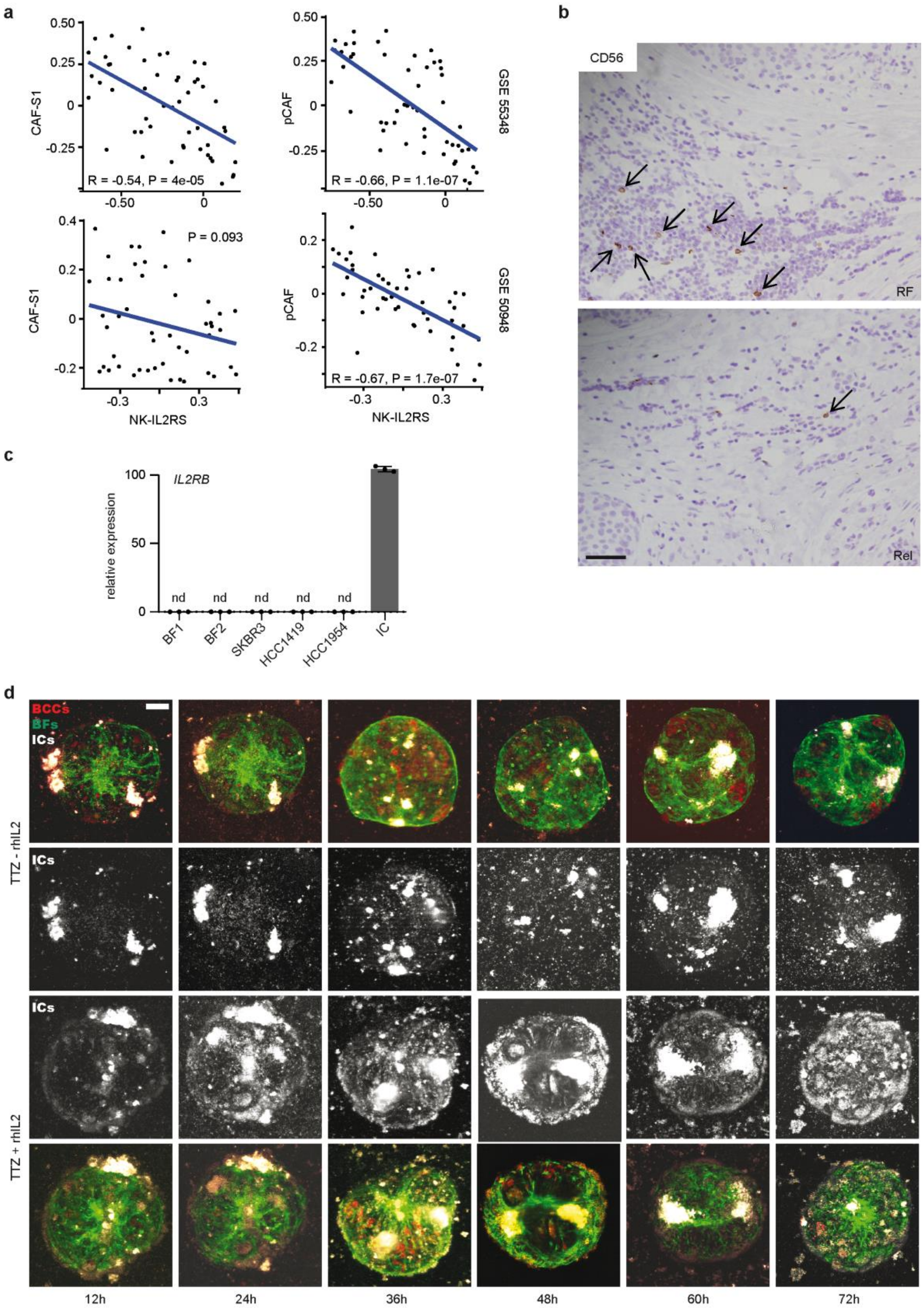
a
CAF-S1
pCAF
R = -0.54, P = 4e-05
R = -0.66, P = 1.1e-07
GSE 55348
P = 0.093
R = -0.67, P = 1.7e-07
GSE 50948
NK-IL2RS
b
CD56
RF
Rel
c
IL2RB
relative expression
nd
BF1
BF2
SKBR3
HCC1419
HCC1954
IC
d
BCCs
BFs
ICs
TTZ - rhIL2
TTZ + rhIL2
12h
24h
36h
48h
60h
72h

Supplementary figure 2. Response to IL2 anti-cancer immunity. (a) Correlations between NK-IL2RS and CAF-S1 or pCAF gene signatures in HER2+ BC patients from GSE50948 and GSE55348 datasets. Correlation coefficients (R) and Spearman *p*-values are indicated. **(b)** Immunostaining of CD56+ cells in Rel and RF HER2+ BC human tumor samples. Arrows point to positive cells. Representative of n=22 tumors. Scale bar: 50 µm. **(c)** qRT-PCR measuring relative expression levels of *IL2RB* gene in the indicated cells cultured *in vitro*. n=3 replicates from 1 out of 2 biologically independent experiments. Values are mean ± s.d. nd: not determined. **(d)** Representative bioimaging over 72 hours follow-up of treated HER2+3DiBCs from figure 3g. n=3. Upper rows: reduced abundance of ICs in HER2+3DiBCs treated with TTZ in absence of rhIL2. Lower rows: increased abundance of ICs in HER2+3DiBCs treated with TTZ in presence of rhIL2. Scale bar: 200 µm. Red: breast cancer cells (BCCs); Green: breast fibroblasts (BFs); White: immune cells (ICs). Rel: relapsing patients; RF: relapse-free patients; TTZ: trastuzumab; rhIL2: recombinant human IL2. Source data are provided as a Source Data file.

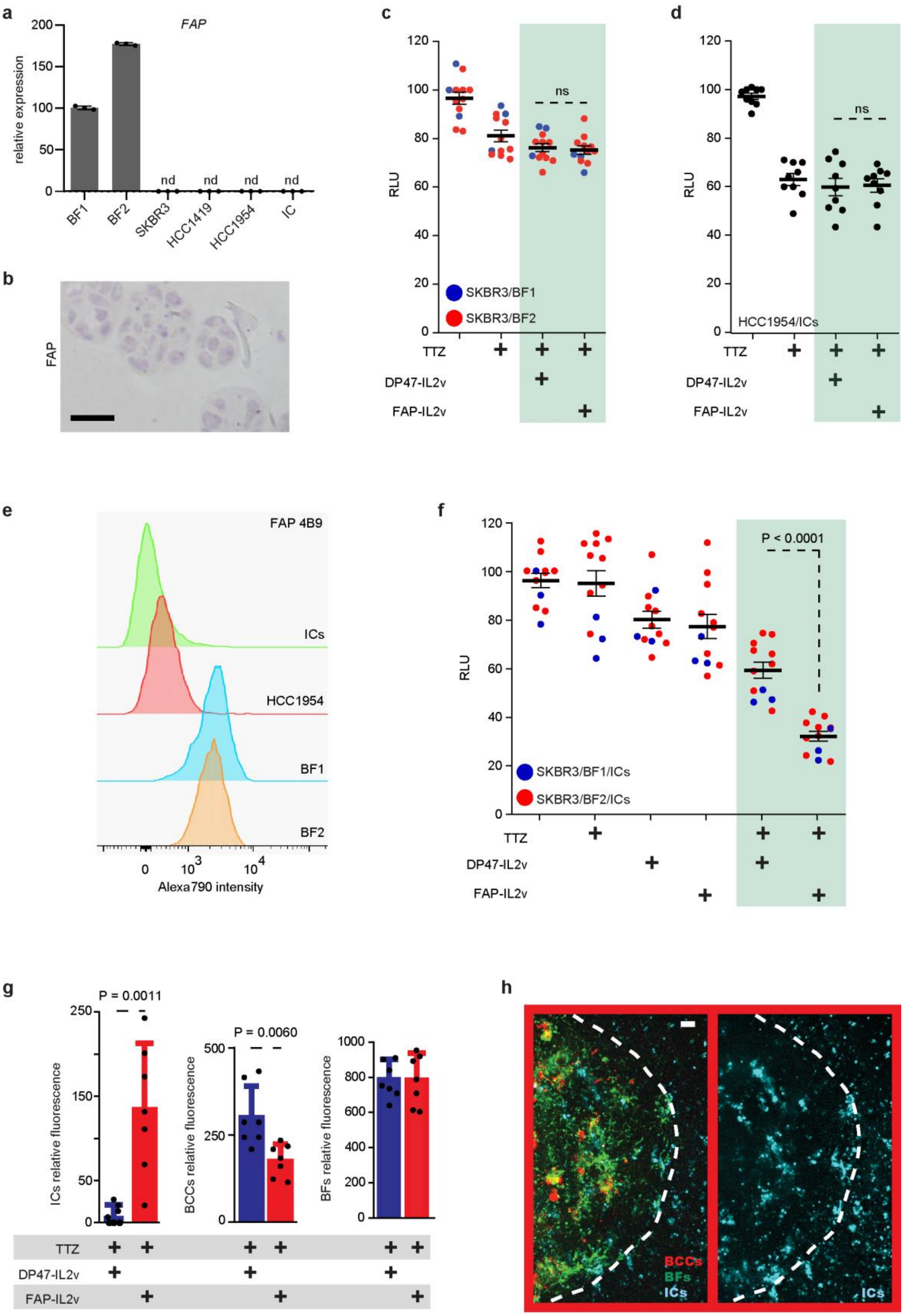
a
FAP
relative expression
200
150
100
50
0
nd
BF1
BF2
SKBR3
HCC1419
HCC1954
IC
b
FAP
c
RLU
ns
SKBR3/BF1
SKBR3/BF2
TTZ
DP47-IL2v
FAP-IL2v
d
HCC1954/ICs
e
FAP 4B9
ICs
HCC1954
BF1
BF2
Alexa790 intensity
f
P < 0.0001
SKBR3/BF1/ICs
SKBR3/BF2/ICs
g
P = 0.0011
P = 0.0060
ICs relative fluorescence
BCCs relative fluorescence
BFs relative fluorescence
h
BCCs
BFs
ICs

Supplementary figure 3. FAP-IL2v restores anti-cancer immunity. (a) qRT-PCR measuring relative expression levels of *FAP* gene in the indicated cells cultured *in vitro*. n=3 replicates from 1 out of 2 biologically independent experiments. Values are mean ± s.d. nd: not determined. **(b)** FAP negative immunodetection in HCC1954 cell culture without BFs. Scale bar: 20 µm. Representative of n=3 independent cell cultures. **(c)** Bioluminescent tracking of SKBR3 in HER2+3DiBCs with BF1 (blue) or BF2 (red) treated as indicated in absence of ICs. IgG1 was used as control for TTZ. n=12 HER2+3DiBCs examined per condition, from 4 biologically independent experiments. Values are mean ± s.e.m. **(d)** Bioluminescent tracking of HCC1954 in HER2+3DiBCs treated as indicated in presence of ICs. IgG1 was used as control for TTZ. n=9 HER2+3DiBCs examined per condition, from 3 biologically independent experiments. Values are mean ± s.e.m. **(e)** Flow cytometry analysis of anti-FAP (4B9 clone) antibody capacity to target ICs, BF1, BF2 or BCCs (HCC1954). Representative of n=2 biologically independent experiments. **(f)** Bioluminescent tracking of SKBR3 in HER2+3DiBCs with BF1 (blue) or BF2 (red) treated as indicated in presence of ICs. IgG1 was used as control for TTZ. n=12 HER2+3DiBCs examined per condition, from 4 biologically independent experiments. Values are mean ± s.e.m. **(g)** Cell-specific fluorescence measurement after 72 hours treatment. TTZ-treated HER2+3DiBCs show increased ICs, decreased HCC1954 and equivalent BFs fluorescence upon FAP-IL2v (red) inoculation compared to control treatment (DP47-IL2v; blue). n=7 biologically independent experiments. Values are mean ± s.d. **(h)** Representative bioimaging of TTZ/FAP-IL2v treated HER2+3DiBCs from (g). Left panel: BCCs/BFs/ICs fluorescence. Right panel: ICs fluorescence alone. Dashed lines delineate tumor inner and outer compartments. Scale bar: 50 µm. Red: breast cancer cells (BCCs); Green: breast fibroblasts (BFs); Cyan: immune cells (ICs); TTZ: trastuzumab; RLU: relative luminescence units. Two-sided, unpaired t-test *p*-values (P) are indicated for (c,d,f,g); ns indicates non-significant. Source data are provided as a Source Data file.

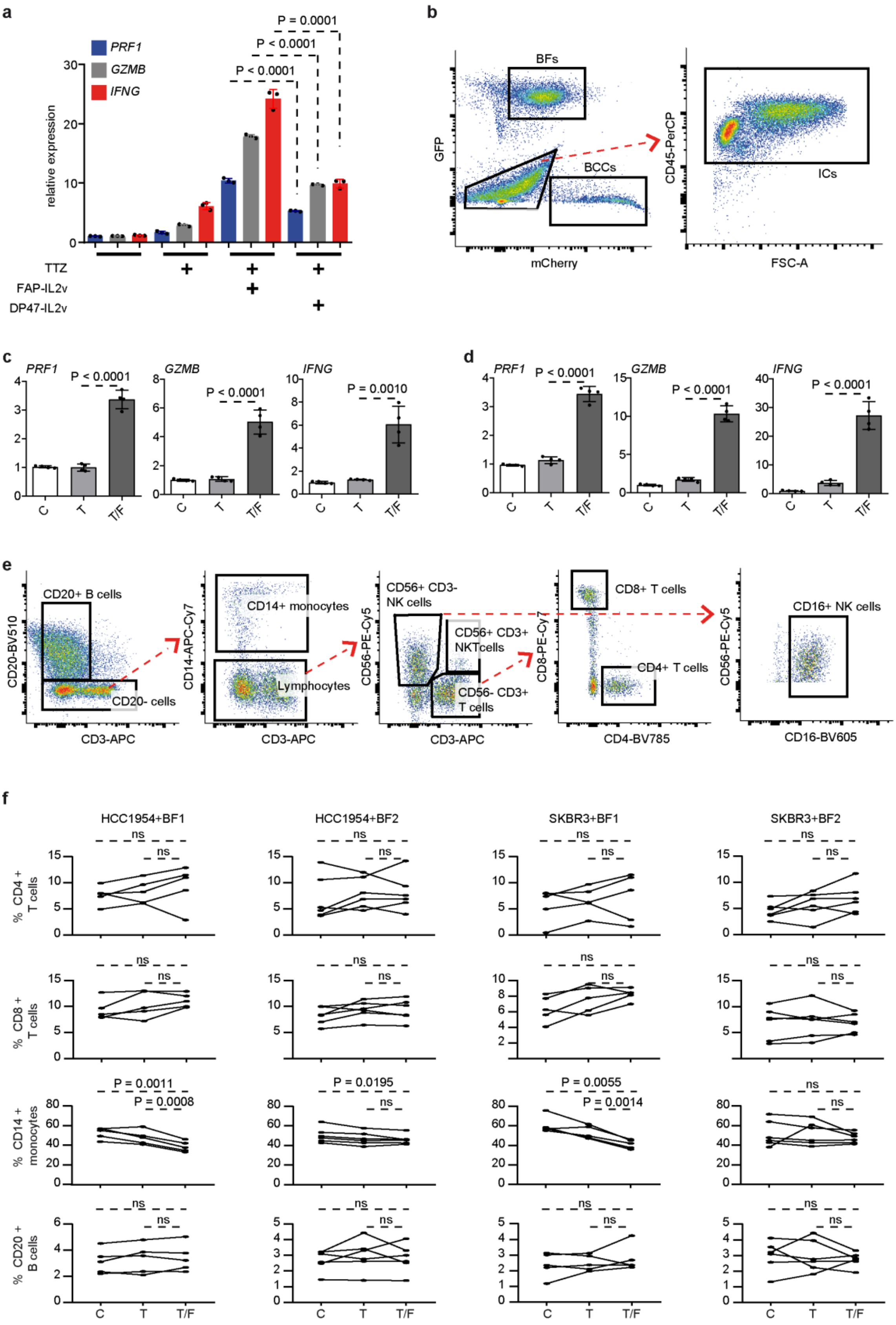
a
PRF1
GZMB
IFNG
relative expression
P = 0.0001
P < 0.0001
P < 0.0001
TTZ
FAP-IL2v
DP47-IL2v
b
BFs
BCCs
GFP
mCherry
CD45-PerCP
ICs
FSC-A
c
PRF1
P < 0.0001
GZMB
P < 0.0001
IFNG
P = 0.0010
C
T
T/F
d
PRF1
P < 0.0001
GZMB
P < 0.0001
IFNG
P < 0.0001
e
CD20+ B cells
CD20- cells
CD20-BV510
CD3-APC
CD14+ monocytes
Lymphocytes
CD14-APC-Cy7
CD56+ CD3-
NK cells
CD56+ CD3+
NKTcells
CD56- CD3+
T cells
CD56-PE-Cy5
CD8+ T cells
CD4+ T cells
CD8-PE-Cy7
CD4-BV785
CD16+ NK cells
CD16-BV605
f
HCC1954+BF1
HCC1954+BF2
SKBR3+BF1
SKBR3+BF2
% CD4 + T cells
% CD8 + T cells
% CD14 + monocytes
% CD20 + B cells
ns
P = 0.0011
P = 0.0008
P = 0.0195
P = 0.0055
P = 0.0014

Supplementary figure 4. FAP-IL2v enhances ADCC through NK activation. (a) Relative gene expression levels of *IFNG* (red), *GZMB* (gray) and *PRF1* (blue) measured by qRT-PCR in HER2+3DiBCs (SKBR3/BF1/IC) treated as indicated. IgG1 was used as control for TTZ. n=3 replicates from 1 out of 2 biologically independent experiments. Values are mean ± s.d. **(b)** Flow cytometry profile illustrating the gating strategy for CD45+ cells purification from HER2+3DiBCs. **(c, d)** Relative gene expression levels of *PRF1*, *GZMB* and *IFNG* measured by qRT-PCR in CD45+ cells purified using gating panels displayed in (b) from HER2+3DiBCs treated as indicated. **(c)**: SKBR3/BF2/IC; **(d)**: HCC1954/BF2/IC. n=4 biologically independent experiments. Values are mean ± s.d. **(e)** Flow cytometry illustrating the gating strategy for infiltrating immune cell subpopulations profiling from HER2+3DiBCs. **(f)** Flow cytometry analyses using gating panels displayed in (e) showing percentages of CD4+ T cells, CD8+ T cells, CD14+ monocytes and CD20+ B cells within infiltrating ICs from HER2+3DiBCs treated with trastuzumab or trastuzumab+FAP-IL2v compared to control (IgG1). n=5 (SKBR3/BF1/IC; HCC1954/BF1/IC) and n=6 (SKBR3/BF2/IC; HCC1954/BF2/IC) biologically independent experiments. C: control, T: trastuzumab, T/F: trastuzumab+FAP-IL2v. Two-sided, unpaired (a,c) and paired (f) t-test *p*-values (P) are indicated; ns indicates non-significant. Source data are provided as a Source Data file.

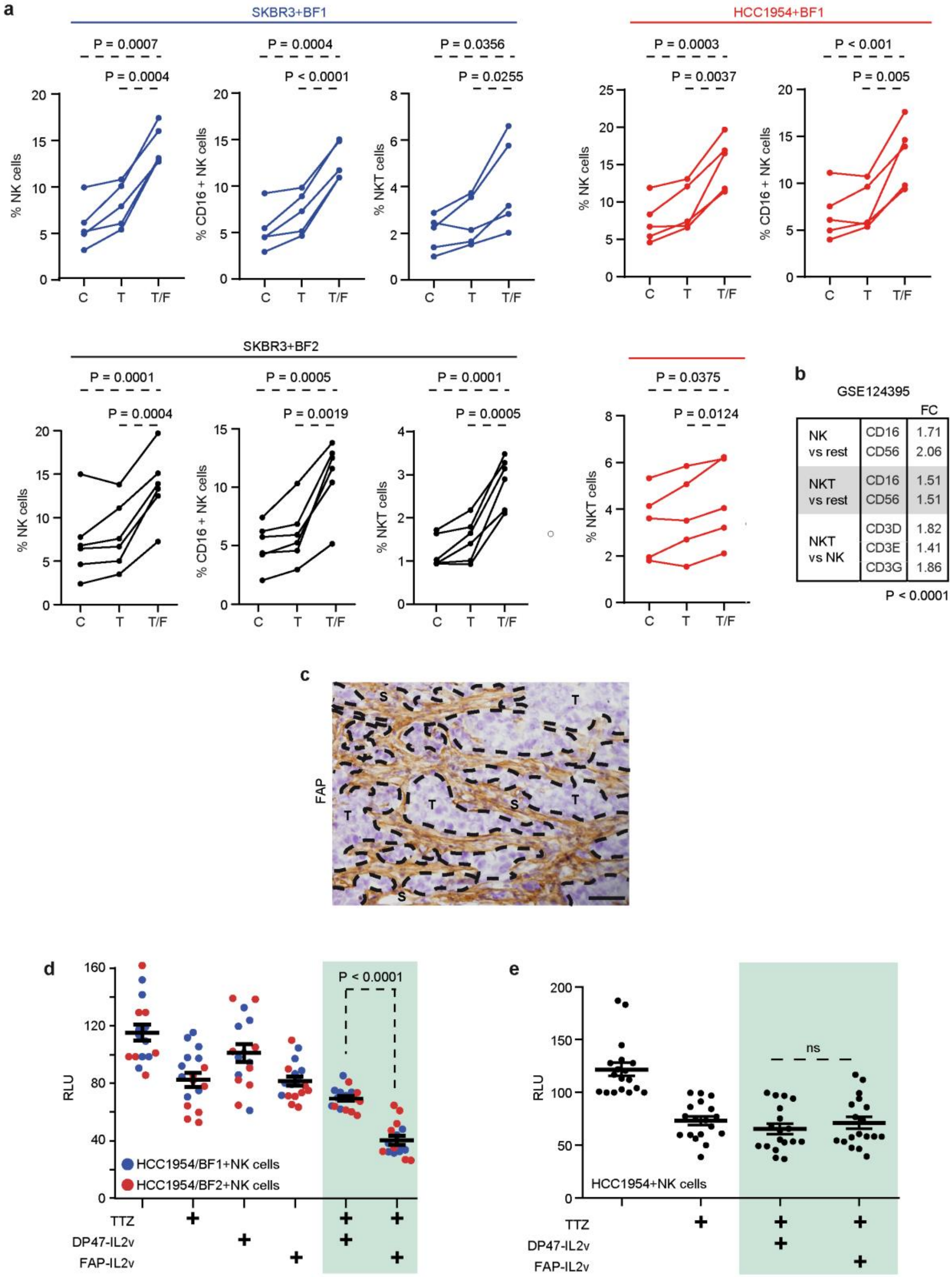
a
SKBR3+BF1
HCC1954+BF1
P = 0.0007
P = 0.0004
P = 0.0004
P < 0.0001
P = 0.0356
P = 0.0255
P = 0.0003
P = 0.0037
P < 0.001
P = 0.005
% NK cells
% CD16 + NK cells
% NKT cells
C
T
T/F
SKBR3+BF2
P = 0.0001
P = 0.0004
P = 0.0005
P = 0.0019
P = 0.0001
P = 0.0005
P = 0.0375
P = 0.0124
b
GSE124395
FC
NK vs rest
CD16 1.71
CD56 2.06
NKT vs rest
CD16 1.51
CD56 1.51
NKT vs NK
CD3D 1.82
CD3E 1.41
CD3G 1.86
P < 0.0001
c
FAP
S
T
d
RLU
P < 0.0001
HCC1954/BF1+NK cells
HCC1954/BF2+NK cells
TTZ
DP47-IL2v
FAP-IL2v
e
ns
HCC1954+NK cells

Supplementary figure 5. FAP-IL2v enhances ADCC through NK activation. (a) Flow cytometry analyses using gating panels from supplementary figure 4e and showing percentages of NK cells, CD16+ NK cells and NKT cells in infiltrating ICs from HER2+3DiBCs treated as indicated. n=5 (SKBR3/BF1/IC, blue; HCC1954/BF1/IC, red) and n=6 (SKBR3/BF2/IC, black) biologically independent experiments. **(b)** NK and NKT gene expression signatures derived from single cell analyses (GSE124395). Upper and middle rows: expression levels of CD56 and CD16 in CD56+CD3- or CD56+CD3+ cells compared to CD56- cells (rest). Lower row: expression levels of CD3D, 3E and 3G in CD56+CD3+ compared to CD56+CD3- cells. **(c)** FAP expression in macroscopic tumor sample derived from subcutaneous injection of HCC1954 into nude mice. T: tumor; S: stroma. Scale bars: 50 µm. Representative of n=6 tumors. **(d)** Bioluminescent tracking of HCC1954 in HER2+3DiBCs with BF1 (blue) or BF2 (red) in presence of NK cells and treated as indicated. IgG1 was used as control for TTZ. n=16 HER2+3DiBCs examined per condition, from 4 biologically independent experiments. Values are mean ± s.e.m. **(e)** Bioluminescent tracking of HCC1954 in HER2+3DiBCs treated as indicated in presence of NK cells. IgG1 was used as control for TTZ. n=18 HER2+3DiBCs examined per condition, from 4 biologically independent experiments. Values are mean ± s.e.m. C: control (IgG1), T: trastuzumab, T/F: trastuzumab+FAP-IL2v; FC: fold change; RLU: relative luminescence units. Two-sided, paired (a) and unpaired (d,e) t-test *p*-values (P) are indicated; ns indicates non-significant. Source data are provided as a Source Data file.

a

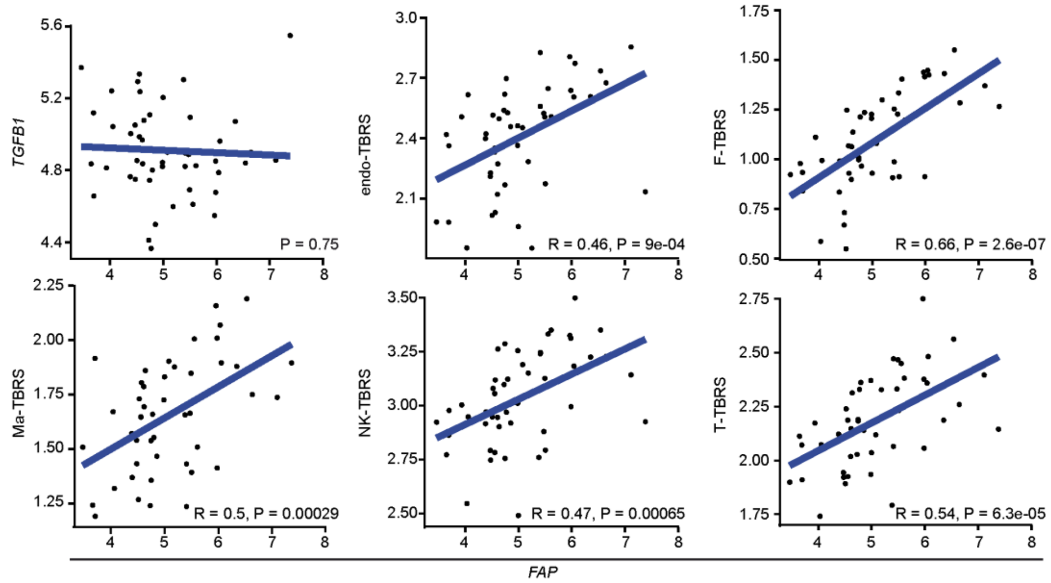

b

GSE	55348	50948
	Endo-TBRS	
ES	-0.179	-0.428
NES	-0.994	-1.458
p-value	0.507798	0.000
FDR	0.48505157	0.0018912825

	55348	50948
	T-TBRS	
ES	0.247	0.294
NES	1.209	0.874
p-value	0.092070095	0.83423424
FDR	0.09504826	0.939998

	55348	50948
	Ma-TBRS	
ES	0.188	0.292
NES	1.154	1.024
p-value	0.005148005	0.2854512
FDR	0.13201034	0.3993195

	55348	50948
	NK-TBRS	
ES	0.188	-0.330
NES	1.031	-1.093
p-value	0.33571428	0.15789473
FDR	0.34881893	0.15987206

c

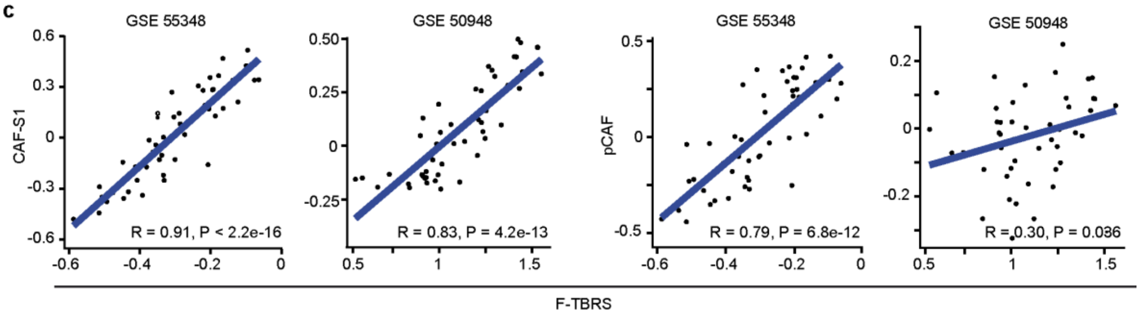

d

		GSE 55348		GSE 50948	
		R	p-value	R	p-value
CAF-S1	Ma-TBRS	0.88	< 2.2e-16	0.63	1.4e-08
	endo-TBRS	0.58	9.4e-06	0.63	2.9e-5
	NK-TBRS	0.77	2.7e-11	0.74	5.9e-15
	T-TBRS	0.74	6.3e-10	0.74	1.3e-11
pCAF	Ma-TBRS	0.82	4.5e-15	0.54	1.3e-04
	endo-TBRS	0.40	2.9e-03	0.11	0.241
	NK-TBRS	0.69	7.7e-12	0.29	4.2e-03
	T-TBRS	0.56	2.9e-06	0.17	0.133

e **f** **g**

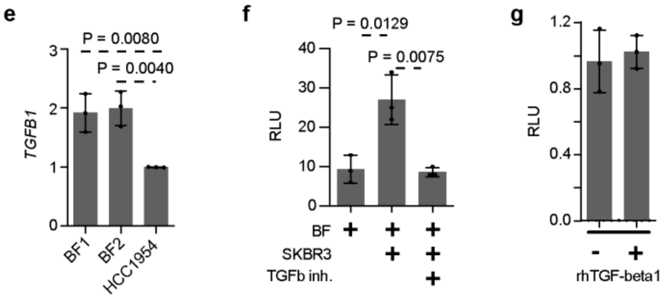

Supplementary figure 6. FAP is a biomarker of TGF-beta activity in BC microenvironment. (a) Correlation between *FAP* mRNA levels and *TGFB1* or TBRSs (From up to down, left to right: *TGFB1*, endothelial-, fibroblast-, macrophage-, NK cell-, T cell- TBRS) in HER2+ BC patients from the GSE50948 dataset. Correlation coefficients (R) and Spearman *p*-values are indicated. **(b)** GSEA of TBRSs comparing pCR vs. RD (GSE50948) and recurring vs. relapse free (GSE55348) HER2+ BC after trastuzumab-based therapy. **(c,d)** Correlation between expression levels of **(c)** CAF-S1 or pCAF and F-TBRS, **(d)** CAF-S1 or pCAF and other TBRSs in HER2+ BC patients from GSE55348 and GSE50948 datasets (From up to down: macrophage-, endothelial-, NK cell-, T cell- TBRS). Correlation coefficients (R) and Spearman *p*-values are indicated. **(e)** Relative gene expression levels of *TGFB1* measured by qRT-PCR in HCC1954, BF1 and BF2. n=3 biologically independent experiments. Values are mean ± s.d. **(f)** Bioluminescent tracking of TGF-beta pathway activation in BFs expressing a luminescent reporter of TGF-beta activity (TGF/SMAD Luciferase Reporter) and cultured as indicated. n=3 biologically independent experiments. Values are mean ± s.d. **(g)** Bioluminescent tracking of TGF-beta pathway activation in HCC1954 expressing TGF/SMAD Luciferase Reporter and cultured with or without rhTGF-beta1. n=3 biologically independent experiments. Values are mean ± s.d. ES: enrichment score; NES: normalized enrichment score; FDR: false discovery rate; RLU: relative luminescence; rhTGF-beta1: recombinant human TGF-beta1. GSEA nominal *p*-value (P) and FDR-adjusted *p*-value are indicated for (b). Two-sided, unpaired t-test *p*-values (P) are indicated for (e,f). Source data are provided as a Source Data file.

Supplementary Table 1: Clinical data from patients treated with anti-HER2-based therapy.

**Characteristics**		
**Age**		
Median		43
Range		29-78
**Sex**		
Female		22
**Primary tumor**		
Breast		22
**Type**		
HER2+		22
**Relapse after treatment**		
Yes		9
No		13
**Metastasis**		
bone		4
Lung		3
liver		2
breast		2
nodal		3
**Treatments**		
Trastuzumab/Pertuzumab		4
Trastuzumab		18
**Treatment length**		
Median (months)		12,5

Supplementary Table 2: Antibodies.

Antibody	Isotype/ Coupling	Catalog-No./ Clone	Manufacturer	Dilution	Reference (PMID)
Anti-β-ACTIN	Mouse monoclonal	AC-74	Sigma-Aldrich	1/30.000	15781629
Anti-FAP	Rat monoclonal	MABS1002	Vitatex	1/1000 (WB) 1/800 (IHC)	23153532
Anti-CD56	Mouse monoclonal	123C3	Dako	1/100	8554110
Anti-FAP	Human IgG	4B9 clone	Roche	1/300 (IF/IHC) 1/100 (FACS)	33974508
Anti-human IIary Ab	Coupled Alex 790™	709-655-149	Jackson Immuno-research	1/500 (IF/FACS)	33817392
Anti-CD45	Mouse monoclonal	IS751	Dako	1/100 (IHC)	21468583
Anti-CD45	Coupled PerCP	304025	BioLegend	1/50 (FACS)	31484076
Anti-CD3	Coupled APC	300411	BioLegend	1/80 (FACS)	33945793
Anti-CD4	Coupled Brilliant Violet 785™	344641	BioLegend	1/50 (FACS)	32923123
Anti-CD8	Coupled PE/Cy7	344711	BioLegend	1/50 (FACS)	31995747
Anti-CD20	Coupled Brilliant Violet 510™	302339	BioLegend	1/50 (FACS)	29892063
Anti-CD16	Coupled Brilliant Violet 605™	302039	BioLegend	1/50 (FACS)	26808670
Anti-CD14	Coupled APC-Cy™7	561709	BD Biosciences	1/50 (FACS)	30629918
Anti-CD56	Coupled PE-Cy™5	561904	BD Biosciences	1/10 (FACS)	25238158
Anti-CD16 blocking Ab	Mouse monoclonal	16-0167-82	eBioscience	1/1000	26014098
