# Supplementary material for: Targeted immunotherapy against distinct cancer-associated fibroblasts overcomes treatment resistance in refractory HER2+ breast tumors
Source: Nat Commun. 2022 Sep 9;13:5310. doi: 10.1038/s41467-022-32782-3 (PMC9463158; doi:10.1038/s41467-022-32782-3)
Supplement: Supplementary file 2 — Reporting Summary [file 41467_2022_32782_MOESM2_ESM.pdf]

nature portfolio

Corresponding author(s): Alexandre Calon

Last updated by author(s): Jul 12, 2022

Reporting Summary

Nature Portfolio wishes to improve the reproducibility of the work that we publish. This form provides structure for consistency and transparency in reporting. For further information on Nature Portfolio policies, see our Editorial Policies and the Editorial Policy Checklist.

Statistics

For all statistical analyses, confirm that the following items are present in the figure legend, table legend, main text, or Methods section.

n/a	Confirmed	
☐	☒	The exact sample size (n) for each experimental group/condition, given as a discrete number and unit of measurement
☐	☒	A statement on whether measurements were taken from distinct samples or whether the same sample was measured repeatedly
☐	☒	The statistical test(s) used AND whether they are one- or two-sided<br>*Only common tests should be described solely by name; describe more complex techniques in the Methods section.*
☐	☒	A description of all covariates tested
☒	☐	A description of any assumptions or corrections, such as tests of normality and adjustment for multiple comparisons
☐	☒	A full description of the statistical parameters including central tendency (e.g. means) or other basic estimates (e.g. regression coefficient) AND variation (e.g. standard deviation) or associated estimates of uncertainty (e.g. confidence intervals)
☐	☒	For null hypothesis testing, the test statistic (e.g. F, t, r) with confidence intervals, effect sizes, degrees of freedom and P value noted<br>*Give P values as exact values whenever suitable.*
☒	☐	For Bayesian analysis, information on the choice of priors and Markov chain Monte Carlo settings
☒	☐	For hierarchical and complex designs, identification of the appropriate level for tests and full reporting of outcomes
☐	☒	Estimates of effect sizes (e.g. Cohen's d, Pearson's r), indicating how they were calculated

Our web collection on statistics for biologists contains articles on many of the points above.

Software and code

Policy information about availability of computer code

Data collection	NIS Elements v.5.30.05, SDS v.2.1, Diva v.9.0
Data analysis	ESTIMATE v.1.0.13, g:Profiler v.2020-10-12, GSEA v.4.1.0, GSVA v.1.45.2, ggplot2 v.3.3.6, Graphpad Prism v.8.0.1, Simplicity v.4.2, SPSS v.22, Imaris v.9.7 (Oxford Instruments) , ImageJ v.1.53i, Qpath v.0.3.2 and Flow Jo v.10.8 software use is detailed in Methods section.

For manuscripts utilizing custom algorithms or software that are central to the research but not yet described in published literature, software must be made available to editors and reviewers. We strongly encourage code deposition in a community repository (e.g. GitHub). See the Nature Portfolio guidelines for submitting code & software for further information.

Data

Policy information about availability of data

All manuscripts must include a data availability statement. This statement should provide the following information, where applicable:

- Accession codes, unique identifiers, or web links for publicly available datasets
- A description of any restrictions on data availability
- For clinical datasets or third party data, please ensure that the statement adheres to our policy

GSE55348, GSE50948, GSE39197 and GSE12198 datasets used in this study are publicly available in the NCBI GEO database.
https://www.ncbi.nlm.nih.gov/geo/query/acc.cgi?acc=GSE50948
https://www.ncbi.nlm.nih.gov/geo/query/acc.cgi?acc=GSE55348
https://www.ncbi.nlm.nih.gov/geo/query/acc.cgi?acc=GSE39197
https://www.ncbi.nlm.nih.gov/geo/query/acc.cgi?acc=GSE12198
The data are available within the Article, Supplementary Information, Supplementary Data and Source Data files provided with this paper.

Field-specific reporting

Please select the one below that is the best fit for your research. If you are not sure, read the appropriate sections before making your selection.

[x] Life sciences   [ ] Behavioural & social sciences   [ ] Ecological, evolutionary & environmental sciences

For a reference copy of the document with all sections, see nature.com/documents/nr-reporting-summary-flat.pdf

Life sciences study design

All studies must disclose on these points even when the disclosure is negative.

Sample size	Sample size was chosen following previous experience in the assessment of experimental variability (Tauriello et al, 2018, PMID: 29443964; Calon et al. 2015, PMID: 25706628).
Data exclusions	When pre-established deteriorating clinical alterations were observed (animal fitness, weight loss), animals were excluded from the analysis. Exclusion occurred prior to treatment initiation and did not lead to data exclusion.
Replication	Generally, all measurements were performed with n>= 3 biological replicates. All attempts at replication were successful.
Randomization	Animals were all females and ranged from 5-6 weeks of age. Mice bearing macroscopic tumors were randomly assigned to experimental groups. For ex vivo experiments, samples were randomized into treatment groups.
Blinding	The investigators were blinded to group allocation and data collection for in vivo experiments. No blinding was applied to ex vivo experiments as each investigator performing a given experiment labeled the corresponding samples and performed the analysis.

Reporting for specific materials, systems and methods

We require information from authors about some types of materials, experimental systems and methods used in many studies. Here, indicate whether each material, system or method listed is relevant to your study. If you are not sure if a list item applies to your research, read the appropriate section before selecting a response.

Materials & experimental systems

n/a	Involved in the study	
[ ]	[x]	Antibodies
[ ]	[x]	Eukaryotic cell lines
[x]	[ ]	Palaeontology and archaeology
[ ]	[x]	Animals and other organisms
[ ]	[x]	Human research participants
[x]	[ ]	Clinical data
[x]	[ ]	Dual use research of concern

Methods

n/a	Involved in the study	
[x]	[ ]	ChIP-seq
[ ]	[x]	Flow cytometry
[x]	[ ]	MRI-based neuroimaging

Antibodies

Antibodies used	Anti-β-ACTIN (WB), Anti-FAP (WB/IHC), Anti-CD56 (IHC), Anti-FAP (IF/IHC/FACS), Anti-human IIary Ab (IF/FACS), Anti-CD45 (IHC), Anti-CD45 (FACS), Anti-CD3 (FACS), Anti-CD4 (FACS), Anti-CD8 (FACS), Anti-CD20 (FACS), Anti-CD16 (FACS), Anti-CD14 (FACS), Anti-CD56 (FACS), Anti-CD16 blocking Ab. Dilutions and applications are detailed in Methods and Supplementary Table 2.

Validation	Anti-β-ACTIN (WB) Mouse monoclonal AC-74 Sigma-Aldrich PMID 15781629<br>Anti-FAP (WB/IHC) Rat monoclonal MABS1002 Vitatex PMID 23153532<br>Anti-CD56 (IHC) Mouse monoclonal 123C3 Dako PMID 8554110<br>Anti-FAP (IF/IHC/FACS) Human IgG 4B9 clone Roche PMID 33974508<br>Anti-human IIary Ab (IF/FACS) Coupled Alex 790TM 709-655-149 Jackson Immunoresearch PMID 33817392<br>Anti-CD45 (IHC) Mouse monoclonal IS751 Dako PMID 21468583<br>Anti-CD45 (FACS) Coupled PerCP 304025 BioLegend PMID 31484076<br>Anti-CD3 (FACS) Coupled APC 300411 BioLegend PMID 33945793<br>Anti-CD4 (FACS) Coupled Brilliant Violet 785TM 344641 BioLegend PMID 32923123<br>Anti-CD8 (FACS) Coupled PE/Cy7 344711 BioLegend PMID 31995747<br>Anti-CD20 (FACS) Coupled Brilliant Violet 510TM 302339 BioLegend PMID 29892063<br>Anti-CD16 (FACS) Coupled Brilliant Violet 605TM 302039 BioLegend PMID 26808670<br>Anti-CD14 (FACS) Coupled APC-CyTM7 561709 BD Biosciences PMID 30629918<br>Anti-CD56 (FACS) Coupled PE-CyTM5 561904 BD Biosciences PMID 25238158<br>Anti-CD16 blocking Ab Mouse monoclonal 16-0167-82 eBioscience PMID 26014098

Eukaryotic cell lines

Policy information about cell lines

Cell line source(s)	BFs were kindly provided by P. Gascon/P. Bragado laboratory (IDIBAPS, Barcelona). Breast cancer cell lines (SKBR3, HCC1419, HCC1954) were provided by the IMIM Cancer Cell Lines Repository (CCLR). Detailed in Cells and cell culture section; Methods.
Authentication	BFs were characterized by measuring the expression of α-SMA and FAP.HER2 upregulation by BCCs was confirmed by RT-qPCR. Detailed in Results section and Cells and cell culture section; Methods.
Mycoplasma contamination	Cell lines were tested weekly for mycoplasma contamination and resulted negative. Detailed in Cells and cell culture section; Methods.
Commonly misidentified lines<br>(See ICLAC register)	No commonly misidentified lines where used in this study.

Animals and other organisms

Policy information about studies involving animals; ARRIVE guidelines recommended for reporting animal research

Laboratory animals	Experiments were performed in pure Swiss nude mice background (Jackson Laboratories). Female 5 to 6 weeks old animals were used. Detailed in Orthotopic Mouse studies section.
Wild animals	No wild animals where used in this study
Field-collected samples	No field-collected samples where used in this study
Ethics oversight	In vivo experiments in murine model were approved by the Animal Research Ethical Committee of Barcelona Biomedical Research Park (CEEA-PRBB; FUE-2018-00801894) and the Catalan government.

Note that full information on the approval of the study protocol must also be provided in the manuscript.

Human research participants

Policy information about studies involving human research participants

Population characteristics	22 women 29 to 78 years old with primary HER2+ breast tumor<br>Relapse after treatment: Yes 9; No 13<br>Metastasis: bone 4; Lung 3; liver 2; breast 2; nodal 3<br>Treatments: Trastuzumab/Pertuzumab 4; Trastuzumab 18<br>Treatment length: Median (months) 12,5
Recruitment	Biological samples were obtained from 22 HER2+ BC patients treated with anti-HER2 therapy. Patients were selected based on HER2+ tumor status, treatment and sample availabilty with no self-selection bias or other biases.
Ethics oversight	Biological human samples were obtained under informed consent and approval of the MarBiobanc Committee (Parc de Salut Mar; 2017/7294/I) according to Ethical regulations. There was no participant compensation. Tumor samples were collected within the usual clinical practice. Clinical information was anonymized by medical doctors collaborating to the project. International standards of Ethical Principles for Medical Research Involving Human subjects (code of ethics, Declaration of Helsinki, Fortaleza, Brazil, October 2013) were followed in accordance with legal regulations on data confidentiality (Organic Law 3/2018 -December the 5th- on the Protection of Personal Data and Digital Rights Guarantee) and on biomedical research (Law 14/2007 -July the 3rd-).

Note that full information on the approval of the study protocol must also be provided in the manuscript.

Flow Cytometry

Plots

Confirm that:

- [x] The axis labels state the marker and fluorochrome used (e.g. CD4-FITC).
- [x] The axis scales are clearly visible. Include numbers along axes only for bottom left plot of group (a 'group' is an analysis of identical markers).
- [x] All plots are contour plots with outliers or pseudocolor plots.
- [x] A numerical value for number of cells or percentage (with statistics) is provided.

Methodology

Sample preparation	In order to investigate the immune infiltrate in HER2+3DiBC, non-infiltrating immune cells were excluded prior analyses as follow. Three DPBS wash were performed after culture media retrieval. BME drops containing the immune infiltrate were then pulled out from the culture well with a cut-off P1000 pipet tip and collected in 500 µl trypsin. Resulting single cells preparation was pooled into cold 10 % FBS medium and processed for immunostaining with anti-CD45, CD3, CD4, CD8, CD20, CD14, CD16, CD56 antibodies mix (see Antibodies section) for 30 min on ice in the dark following manufacturer's protocol. For FAP analyses, single cells suspension of either BFs, BCCs or ICs were incubated with anti-FAP 4B9 antibody for 30 min on ice. Detection was achieved with Alexa 790-coupled secondary anti-human antibody (see Antibodies section) following manufacturer's protocol. Cells were washed in 2 ml of cold 2 % FBS DPBS and centrifuged at 400 g/ 10 min/ 4 °C. Cell pellets were resuspended in 2 % FBS DPBS. Cell suspension was stained with DAPI. Detailed in Cell sorting section; Methods.
Instrument	Cell sorting and analysis was performed in a FACSAria II SORP cytometer (BD Biosciences). Detailed in Cell sorting section; Methods.
Software	Data analysis was performed with Flow Jo software. Detailed in Cell sorting section; Methods.
Cell population abundance	Compensation was performed using negative control samples and single positive controls. Post-sort analysis was performed to determine the purity of sorted cells prior to RNA extraction. Abundance of relevant cell populations within post-sort fractions are provided in supplementary table 5. Detailed in Cell sorting section; Methods.
Gating strategy	In all flow cytometry experiments, initial gatings were performed as follow: 1) FSC-A/SSC-A: debris exclusion; 2) FSC-A/DAPI: viable cells selection; 3) FSC-A/FSC-H: single cells selection; 4) GFP/mCherry: selection of BCs and BFs; 5) FSC-A/CD45: leukocytes selection (supplementary figure 4b). Further immune cells identification performed from step 5) are 6) Anti-CD3 Coupled APC/Anti-CD20 Coupled Brilliant Violet 510TM, 7) Anti-CD3 Coupled APC/Anti-CD14 Coupled APC-CyTM7, 8) Anti-CD3 Coupled APC/Anti-CD56 Coupled PE-CyTM5, 9) Anti-CD4 Coupled Brilliant Violet 785TM/Anti-CD8 Coupled PE/Cy7, 10) Anti-CD16 Coupled Brilliant Violet 605TM/Anti-CD56 Coupled PE-CyTM5 (described in supplementary figure 4e).

- [x] Tick this box to confirm that a figure exemplifying the gating strategy is provided in the Supplementary Information.
