# Supplementary material for: Targeted immunotherapy against distinct cancer-associated fibroblasts overcomes treatment resistance in refractory HER2+ breast tumors
Source: Nat Commun. 2022 Sep 9;13:5310. doi: 10.1038/s41467-022-32782-3 (PMC9463158; doi:10.1038/s41467-022-32782-3)
Supplement: Supplementary file 3 — Description of Additional Supplementary Files [file 41467_2022_32782_MOESM3_ESM.pdf]

**Title:** Supplementary Data 1:
**Description:** Gene signatures by gene names

**Title:** Supplementary data 2:
**Description:** Gene signatures by illumina probes

**Title:** Supplementary data 3:
**Description:** Gene signatures by affymetrix probes

**Title:** Supplementary data 4:
**Description:** Single gene enrichments in nonresponding vs responding patients.

**Title:** Supplementary data 5:
**Description:** Cell subtypes abundance in treated HER2+3DiBC
